# Knowledge and skills of pre-eclampsia management among healthcare providers working in antenatal clinics in Zanzibar

**DOI:** 10.1186/s12913-022-08892-5

**Published:** 2022-12-12

**Authors:** Saada Ali Seif, Salma Ali Rashid

**Affiliations:** 1 Department of Nursing Management and Education, The University of Dodoma, P.O.BOX 259, Dodoma, Tanzania; 2 Department of Clinical Nursing, The University of Dodoma, P.O.BOX 259, Dodoma, Tanzania

**Keywords:** Pre-eclampsia, Management, Skills, Knowledge, Healthcare providers, Antenatal clinic, Predictors, Zanzibar

## Abstract

**Background:**

Pre-eclampsia and eclampsia are the leading causes of perinatal morbidity and mortality worldwide. Early detection and treatment of preeclampsia is lifesaving; however, evidence suggests that the majority of women in low and middle income-countries are not routinely screened for high blood pressure during antenatal care, that those with severe and mild pre-eclampsia are not monitored for blood pressure and proteinuria as needed, and the magnesium sulphate is not administered as needed. The purpose of this study was therefore to assess knowledge and skills in pre-eclampsia and eclampsia management and their associated factors among healthcare providers working in antenatal clinics in Zanzibar.

**Methods:**

This was a cross-sectional analytical study conducted in all levels of healthcare facilities in Zanzibar. The study involved 176 healthcare providers (nurses and doctors) who were randomly selected. A self-administered questionnaire was used to collect data and descriptive and inferential statistics were used in the analysis whereby logistic regression models were employed. The Chi-square coefficient, odds ratio, and 95% confidence intervals were reported, and the level of significance was set at *p* < 0.05.

**Results:**

The mean age of healthcare providers was 35.94 (SD ± 7.83) years. The proportion of healthcare providers with adequate knowledge was 49.0%, and 47% had adequate skills. Knowledge level was predicted by working in higher healthcare facility levels (AOR: 3.28, 95% CI: 1.29–8.29), and having attended on-the-job training on pre-eclampsia (AOR: 7.8, 95% CI: 2.74 − 22.75). Skills were predicted by having attended on-job training (AOR: 8.6, 95% CI: 2.45 − 30.16), having working experience of five years or above in antenatal care units (AOR: 27.89, 95% CI: 5.28 − 148.89) and being a medical doctor or assistant medical doctor (AOR: 18.9, 95% CI: 2.1–166).

**Conclusion:**

Approximately half of Zanzibar’s ANC healthcare workers demonstrated inadequate knowledge and skills in preeclampsia care, indicating a critical need for targeted interventions to reduce maternal morbidity and mortality. Knowledge is predicted by attending on-the-job training and working in higher healthcare facility level, while skills is predicted by attending on job training, more years of working experience in antenatal care units and being a medical doctor or assistant medical doctor The study recommends the healthcare facility institutions to provide on-the-job training to for the healthcare providers working in lower healthcare facility levels.

**Supplementary Information:**

The online version contains supplementary material available at 10.1186/s12913-022-08892-5.

## Background

Hypertensive Disorders Pregnancy (HDP), including Pre-eclampsia and Eclampsia (PE/E) are the leading causes of perinatal morbidity and mortality worldwide, [1]  accounting for 10–15% of direct maternal deaths. [2]. The prevalence of pre-eclampsia in developing countries ranges from 1.8 to 16.7%. [3–6, 3]  In Tanzania, the prevalence of pre-eclampsia is 4.2% [7] and in Zanzibar, the prevalence among prenatal women is 9% [8] .

It was estimated that PE/E-related maternal deaths would be reduced by at least 84% in Low-and Middle-Income Countries (LMICs) if there was an early detection of proteinuria and hypertension in pregnancy, timely treatment of severe pre-eclampsia and eclampsia with magnesium sulphate, and early delivery of the fetus. [9] However, poor knowledge and skills about PE/E and its management among healthcare providers, accompanied by inadequate supplies, cause a significant number of women to continue suffering from HDP without receiving proven lifesaving interventions, and this contributes to the burden of HDP in LMICs [10–12]. This accounts for the third delay, which is a delay in receiving adequate health care in the three-delay model for receiving appropriate and timely management. For instance, studies from LMICs reported that, contrary to World Health Organization (WHO) guidelines, women were not regularly screened for high blood pressure during Antenatal Care (ANC), [13]. Moreover, not all women with severe and mild pre-eclampsia had their blood pressure and proteinuria monitored as needed [14] and not all women received magnesium sulfate as required [14, 15].

It has also been reported that various cadres of health care providers (doctors, nurses, midwives, and community care providers) have limited knowledge and skills regarding screening of PE/E management in LMICs [16–18]

## Methods

### Study design and study setting

This study employed an analytical cross-sectional study design and it was conducted in Zanzibar, which is a semi-autonomous state of Tanzania, in the year 2021. In total, there are 154 healthcare facilities at primary level (122 Primary Health Care Units (PHCU), 34 Primary Health Care Units Plus (PHCU +) and 4 Primary Health Care Centre or Cottage (PHCC). There are three secondary healthcare facilities (three district and one regional hospitals) and one tertiary level (referral/national hospital). Healthcare facilities of all levels provide antenatal care services to pregnant women. The number of healthcare providers working in ANC units in all healthcare facilities is 563.

### Study population and sample size

The study population was all healthcare providers working in ANC units. All healthcare providers approached to participate in this study agreed to participate. The study included nurses and medical doctors who were willing to participate. The sample size was calculated by using the Cochran formula, [21] which is *n* = Z^2^P(1-P)/e^2^, where: *n* = minimum sample size, Z score value = 1.96 for 95% confidence level, *P* = proportion of healthcare providers with knowledge of pre-eclampsia, which is 11.8%, [22] and e = acceptable margin of error, which is 5%. After adding 10% of non-response, the total sample size for this study was 176.

### Sampling procedure

The censor method was used to select health centers, district hospitals, regional hospital, and referral hospitals, while a systematic random sampling method was used to select 46 PHCUs. Furthermore, the number of healthcare providers per each selected healthcare facility level was obtained through proportionate sampling using the formula ni = (Ni/Nt)*n, [23] where t ni = the tnumber of health care providers needed from each facility, Ni = the total number of health care providers working in ANC from each healthcare facility, Nt = the total number of health care providers working in ANC in all selected facilities, and n = the estimated sample size. The sample selection of healthcare providers in tertiary hospital was 11 out of 35, secondary hospitals were 19 out of 69, and primary hospitals were 146 out of 467. Within the healthcare facility, all healthcare providers who were willing to participate were selected using simple random sampling.

### Data collection methods and tools

Data was collected using a self-administered questionnaire using a pre-tested standardized structured questionnaire. The tool for assessing knowledge on pre-eclampsia management was adopted from Olaoye et al. [24]. The tool has ten multiple choice questions. The Cronbach alpha was 0.72, and for this study the Cronbach alpha was 0.7. The tool for assessing skills in the management of pre-eclampsia was adopted from the standardized structured questionnaire with seven questions of scenario in a dichotomous (yes/no) format adopted from Jhpiego, [25]. After pre-test, the tool had a Cronbach’s alpha of 0.88.

### Measurement of variables

Ten items on signs and symptoms, risk factors, appropriate drugs and dose, and ways to control convulsions and magnesium toxicity were used to assess knowledge of pre-eclampsia management (Appendix Addition file 1). One point was awarded for the correct answer and zero points for the incorrect answer. The total score was 10 points, and those who scored 80% and above of the total points were considered as having adequate knowledge.

Pre-eclampsia management skills were measured by 7 items of scenario questions in which the participant was required to mention all the important steps in carrying out a certain procedure in relation to the management of pre-eclampsia according to the scenario. e.g. Magnesium sulphate is given at 4 gm intravenous diluted in 20 cc of saline, given over 15–20 min, followed by 5 g intramuscularly every 4 h on alternate buttocks for 24 h (appendix additional file 2). One point was awarded for the completed mentioned steps of the full scenario, and zero points were awarded for not completed steps in the scenario. The total score was 7 and those who scored 80% or above of the total points were considered as having adequate skills.

### Data analysis

Analysis of the data was performed using SPSS software version 25. Descriptive statistics were used to describe the background characteristics of study participants and their level of knowledge and skills. A chi-square test was used to determine the relationship between variables, and a logistic regression model was used to determine the predictors of knowledge and skills. The predictors that were included in the model were the background characteristics of healthcare providers, which included social demographic characteristics, work and professional-related characteristics. The sole effect of each variable was determined by controlling for the effect of other background characteristics that were found significant in the simple logistic regression model. The 95% confidence interval (CI) and odds ratio were reported and the p-value of < 0.05 was considered as the statistical significance difference.

## Results

### Social demographic characteristics of healthcare providers

The mean age of healthcare providers was 35.94 (SD ± 7.83) years. About half 100(56.8%) were female, 71(40.3%) were working at PHCU+, 96(54.5%) were nurses with diploma qualification, and 101(56.7%) had never attended any on-the-job training related to pre-eclampsia Table 1.

**Table 1:** Demographic Characteristic of Healthcare providers (*N* = 176)

Variable	Number	Percentage
**Age group (years)**		
Under 30	37	21.0
30–39	91	51.7
40–49	34	19.3
50 and older	14	8
**Sex**		
Male	76	43.2
Female	100	56.8
**Professional qualification**		
Diploma in nursing	96	54.5
Degree in nursing	10	5.7
Master in Nursing	3	1.7
Clinical officer	47	26.7
Assistant medical officer	2	1.1
Medical doctor	18	10.2
**Working Facility level**		
PHCU	47	26.7
PHCU+	71	40.3
Health center (PHCC)	28	15.9
District hospital	14	8
Regional hospital	5	2.8
Tertiary hospital	11	6.3
**On job training related to management of pre-eclampsia**		
Attended training	75	46.6
Not attended training	101	57.4
**Experience in ANC (years)**		
Less than 5	88	50
5 and above	88	50

### Healthcare providers’ knowledge of PE/E management

Results showed that all 176(100%) healthcare providers were able to correctly define pre-eclampsia, correctly identify tests for prediction of pre-eclampsia, and correctly identify signs and symptoms of pre-eclampsia. A majority, i.e., 138(78.4%) of the healthcare providers could correctly identify the stated drugs for controlling blood pressure and a large majority, i.e., 147(83.5%) of them could correctly identify the categories of pre-eclampsia that could be treated using Magnesium Sulphate (Table 2). The total knowledge score was 10 (Min = 4, Max = 10). Eighty-six (49.0%) healthcare providers were found to have adequate knowledge on PE/E management of.

### Healthcare providers skills on PE/E management

The item analysis of participants in assessing their skills revealed that less than half 82(44.4%) of healthcare providers could correctly describe the screening of danger signs procedure, 82(46.6) could correctly describe the procedure of assessing convulsion, and 64(36.4%) could correctly describe the steps of administering magnesium sulphate loading dose (Table 2). The total skill score was 7 (Min = 1, Max = 7). Eighty-three (47.0%) healthcare providers were found to have adequate skills in the PE/E management.

**Table 2:** Healthcare providers responses to the question related to knowledge and skills on the PE/E management

Questions	Correct n(%)	Incorrect n(%)
**Knowledge**		
What is pre-eclampsia?	176(100)	0(0.0)
At what week of gestation does pre-eclampsia develop?	138(78.4)	38(21.6)
What are the symptoms of pre-eclampsia?	176(100)	0(0.0)
What is the screening test for the prediction of pre-eclampsia?	176(100)	0(0.0)
What are the risk factors for developing pre-eclampsia?	124(70.5)	52(29.5)
What are the stated drugs for controlling blood pressure?	138(78.4)	38(21.6)
In which category of pre-eclampsia doe’s magnesium sulphate injection be used?	147(83.5)	29(16.5)
What is the loading dose of magnesium sulphate?	88(50)	88(50)
What are the signs of magnesium sulphate toxicity?	111(63.5)	65(36.9)
What is the antidote to magnesium sulphate toxicity?	159(90.3)	17(9.7)
**Skills**		
Describe screening of danger signs procedure	82(46.6)	94(53.4)
Describe steps of giving the loading dose of magnesium sulphate	64(36.4)	112(63.6)
Describe the steps of giving maintenance dose of magnesium sulphate	65(36.9)	111(63.1)
Describe the steps of assessing magnesium sulphate toxicity	59(33.5)	117(66.5)
Describe the steps of giving antidote of magnesium sulphate	98(55.7)	78(44.3)
Describe the steps of checking urine for protein	176(100)	0(0.0)
Describe the steps of assessing for convulsion	82(46.6))	94(53.4)

### Bivariate and multivariate logistic regression models for predictors of knowledge of PE/E management

A cross-tabulation was conducted to determine factors that have a significant relationship with knowledge. The findings revealed that knowledge of pre-eclampsia management was significantly associated with age (*p* < 0.0001), healthcare facility level (*p* < 0.0001), professional qualification level (*p* < 0.0001), on-the-job training on pre-eclampsia (*p* < 0.0001) and working experience in ANC units (*p* < 0.0001) **(**Table 3).

The binary logistic regression results revealed an association between respondent aged 40 t to 49 (OR = 11.9, *p* = 0.001), and those aged 50 and up (OR = 27.7, *p* < 0.001) and knowledge. Other factors were working in a higher level healthcare facility (OR = 3.4, *p* < 0.001), attending pre-eclampsia on-the-job training (OR = 20.8, *p* < 0.001), and having more than 5 years of working experience at an ANC (OR = 7.9, *p* < 0.001). In a multivariate logistic regression model, the sole effect of each factor was determined, and the result revealed that healthcare providers who worked in higher healthcare facility levels were 3.2 times more likely to have adequate knowledge compared to those who worked in lower healthcare facility levels (AOD: 3.28, 95%CI 1.29–8.29). Moreover, the healthcare providers who had attended pre-eclampsia on-the-job training were 7.8 times more likely to have adequate knowledge compared to those who had not attended (AOD: 7.8, 95%CI 2.74 − 22.75). Other variables did not show an independent association with knowledge (Table 3).

**Table 3:** Bivariate and multivariate logistic regression models for the predictors of knowledge of PE/E management (*N* = 176)

Variables	Knowledge Level				
	**Total**	**Adequate knowledge** **n(%)**	**COR (95% CI)**	**AOR (95% CI)**	**p-value**
**Age group (years)** ^a^					
Under 30	37	7(18.9)	Ref.		
30–39	91	42(46.2)	3.67(1.4–9.2)	1.18(0.3–3.5)	0.76
40–49	34	25(73.5)	11.90(3.8–36.5)^b^	1.57(0.3–7.3)	0.56
50 and older	14	12(87.5)	25.7(4.6-141.9)^b^	3.19(0.3–26.0)	0.28
**Sex**					
Male	76	39(51.3)	-	-	-
Female	100	47(47)	-		
**Working facilities level**,^**a**^					
Primary level	118	46(39.0)	Ref.		
Secondary and tertiary level	58	40(69)	3.47(1.7–6.7)^b^	3.28(1.2–8.2)	0.01
**Professional qualification** ^a^					
Diploma in nursing	96	30(31.2)	Ref		
Degree/master in nursing	13	13(100)	355(0.00)	-	-
Clinical officer	47	23(48.9)	2.1(1.0-4.3)	-	-
Assistant/medical doctor	20	20(100)	355(0.00)	-	-
**Pre-eclampsia on job training** ^a^					
Never attended training	101	22(21.7)	Ref.		
Attended training	75	64(85.3)	20.89(9.4–46.2)^b^	7.8(2.7–22.7)	< 0.001
**Experience in ANC (years)** ^a^					
Less than 5	88	22(25.0)	Ref.		
5 and above	88	64(72.7)	8(4.1–15.6)^b^	2.3(0.7–7.1)	0.15

^a^*p*-value < 0.05 in chi square test

^b^*p*-value < 0.05 in simple logistic regression

### Bivariate and multivariate logistic regression models for predictors of skills on PE/E management

The results of cross-tabulation revealed that skills in management of pre-eclampsia were significantly associated with age (*p* < 0.001), working healthcare facility level (*p* = 0.005), professional qualification (*p* < 0.005), attending pre-eclampsia on-the-job training (*p* < 0.001) and working experience in ANC (*p* < 0.001) (Table 4).

When a binary logistic regression model was run, results revealed that being in age over of the age of 30 years, being a medical doctor (OR = 8.2, *p* < 0.001), attending pre-eclampsia on job training (OR = 55.6, *p* < 0.001) and working experience of five years and above in ANC (OR = 37.9, *p* < 0.001) were statisticaly significant associated with skills. When a multivariate logistic regression model was applied, which allows the control for the effect of other confounder variables, the results revealed that healthcare providers who were medical doctors or assistant medical doctors had 18.9 times higher odds of having adequate skills compared to nurses with a diploma level of qualification (AOR: 18.9, 95% CI: 2.1–166, *p* = 0.008). Furthermore, healthcare providers who had attended pre-eclampsia on-the-job training were 8.6 times more likely to have adequate skills compared to those who had not attended the training (AOD: 8.6, 95% CI: 2.45 − 30.16, *p* < 0.001). Moreover, those healthcare providers with working experience of five years or above in ANC were 27.8 times more likely to have adequate skills compared to those with less than five years experience in ANC (AOD: 27.89, 95%CI 5.28 − 148.89, *p* < 0.001) (Table 4).

**Table 4:** Bivariate and multivariate logistic regression models for the predictors of skills in the PE/E management (*N* = 176)

Variables	Skills Level				
	**Total**	**Adequate skills** **n(%)**	**COR (95% CI)**	**AOR (95% CI)**	**p-value**
**Age group (years)** ^a^					
20–39		41(32.5)	12.5(5.2–30.4)	2.8(0.7–15.6)	0.15
40–59		42(85.7)	Ref		
**Sex**					
Male	76	37(48.5)	-	-	
Female	100	46(46)	-	-	
**Working facilities level** ^a^					
Primary level	118	47(39.8)	**Ref.**		
Secondary and tertiary level	58	36(62.1)	2.47(1.2–4.7)^b^	1.7(0.3–9.6)	0.5
**Professional qualification** ^a^					
Diploma in nursing	96	39(40.6)	**Ref**		
Degree/master in Nursing	13	12(92.3)	17.5(2-140)	12(0.5–251)	0.11
Clinical officer	47	15(31.9)	0.6(0.3–1.4)	0.4(0.1–1.7)	0.44
Assistant/medical doctor	20	17(85)	8.2(2.2–30.1)^b^	18.9(2.1–166)	0.008
**Pre-eclampsia on job training** ^a^					
Never Attended training	101	15(14.9)	**Ref.**		
Attended training	75	68(90.7)	55.6(21.4–144)^b^	8.7(2.7–27.5)	< 0.001
**Experience in ANC (years)** ^a^					
Less than 5	88	10(11.4)	Ref.		
5 and above	88	73(83.0)	37.9(16-89.8)^b^	27.9(5.2–148)	< 0.001

^a^*p*-value < 0.05 in chi square test

^b^*p*-value < 0.05 in simple logistic regression

## Discussion

The current study was conducted in ANC units of all healthcare facility levels in Zanzibar to understand whether healthcare providers working in this setting have the knowledge and skills to support early diagnosis and management of pre-eclampsia. This may indicate the type of care currently available in this setting, and this information will help in investigating whether interventions to healthcare providers are urgently needed and whether we’re meeting the WHO goals.

According to the findings of this study, less than half of healthcare providers have adequate (49%) knowledge of pre-eclampsia management This low proportion is partly contributed by a lack of on-the-job training on the management of pre-eclampsia among healthcare providers working in ANC units. On-the-job training was shown in this study to strongly predict the high knowledge level, and since the majority of healthcare providers did not attend any training (57.4%), this implies the low knowledge level observed. Moreover, there is an unequal proportion of those who attended training between those working in lower (primary) and higher (secondary and tertiary) healthcare facilities. This study shows that only 26.8% of healthcare providers from lower healthcare facilities attended on-the-job training on management of pre-eclampsia, and this may reflect the type of services that are provided at this level of care, where the majority of pregnant women have their first contact. This finding suggests that the capacity building of healthcare providers at lower facilities levels is minimum.

Furthermore, working in a higher level of healthcare facility has been shown to predict a high knowledge level. This was expected because usually the higher healthcare facility levels are where professionals with high qualifications are allocated (Ministry). This is because these facilities are the referral facilities where complicated cases are referred to for more specialized management. Therefore, working there creates more opportunities for exposure of cases and gaining experience in their management and thus improves healthcare providers’ knowledge. This finding is similar to what was reported in India, [26]. Pakistan, [11] and Zambia [18] that, healthcare providers from higher healthcare facilities level were more knowledgeable about the management of pre-eclampsia as compared to those from lower healthcare facilities level.

.On the contrary, the finding from this study of 49% differs from what was reported in southern Nigeria [24] that the knowledge level was 64.2%, The difference might be due to differences in study settings and sample characteristics. The current study was conducted in facilities of all levels of care and involved all healthcare providers, including nurses and medical doctors, while the former studies were conducted at secondary level [24]. The facility level, professional qualifications, and on-thejob training were observed in this study to have an association with the knowledge level of healthcare providers, and this could be the reason for the difference observed.

Appropriate skills in the management of maternal conditions are critical to avoid delays in life-saving at the health facility [27]. Findings from this study reveal that less than half of healthcare providers (47.16%) had adequate skills in the management of pre-eclampsia. This low proportion is partly contributed by the lack of on-the-job training and less working experience in the ANC unit. Healthcare providers, skills in this study were shown to increase through more years of experience in ANC and through attending training on the management of pre-eclampsia. Experience improves skills through more exposure and management in many cases, while training improves skills by refreshing what is already known in practice, assisting in correcting the practical errors and providing new and advance technique of practice. This finding is comparable to what is reported in Afghanistan [28] which reported that healthcare providers who received training related to management of pre-eclampsia, and exposed to more cases of severe pre-eclampsia proved to have stronger pre-eclampsia management skills. The imitations of this study is on the skills measurement, this study relied on subjective assessment rather than objective assessment, this may lea to over estimation of skills scores. Therefore, future studies should consider the observation of actual practice to assess the skills either in real clinical stings or in a simulation environment.

## Conclusion

Approximately half of Zanzibar’s ANC healthcare workers demonstrated inadequate knowledge and skills in preeclampsia care, indicating a critical need for targeted interventions to reduce maternal morbidity and mortality. Knowledge is predicted by attending on-job training and working in higher healthcare facility levels, while skills is predicted by being a medical doctor or assistant medical doctor, on-the-job training and more years of working experience in ANC units. There is a huge gap in knowledge and skills between healthcare providers working in lower and higher healthcare facility levels. This may impair the services in lower healthcare facilities l where the majority of pregnant women first seek services. This may be lead to the delay in receiving the appropriate management, the third delay in three delay model, which may contribute to poor maternal outcome. This study recommends the health facility institutions to provide on-the- job training to the healthcare providers working in lower healthcare facility levels and to carefully plan the periodic reshuffling of healthcare providers by discouraging the reshuffling from ANC units to other departments so that they may gain more experience in ANC units, and encourage the reshuffling between healthcare facility levels (e.g. from lower level to high level) so that they can learn from others.

## Supplementary Information

**Additional file 1.**

## Data Availability

The dataset supporting the conclusions of this article is available from the corresponding author on reasonable request.

## Abbreviations

ANC: Antenatal Care
HDP: Hypertensive Disorders in Pregnancy
LMICs: Low-and-Middle Income Countries
PE/E: Pre-eclampsia and eclampsia
PHCU: Primary Health Care Units
PHCU +: Primary Health Care Units Plus
PHCC: Primary Health Care Cottage
WHO: World Health Organization
