# Supplementary material for: Knowledge and skills of pre-eclampsia management among healthcare providers working in antenatal clinics in Zanzibar
Source: BMC Health Serv Res. 2022 Dec 12;22:1512. doi: 10.1186/s12913-022-08892-5 (PMC9746160; doi:10.1186/s12913-022-08892-5)
Supplement: Supplementary file 1 — Additional file 1. [file 12913_2022_8892_MOESM1_ESM.docx]

Appendix 1

Questionnaire of healthcare providers for assessing knowledge and skills of the management of pre-eclampsia

Name of facility …………………….

Working Facility level

1. PHCU ( )
2. PHCU+ ( )
3. Health center ( )
4. District hospital ( )
5. Regional hospital ( )
6. Tertiary hospital ( )

**Part one: Demographic characteristics of HCP**

1. What is your professional qualification?

1. Clinical officer ( )
2. Assistant Medical Officer ( )
3. Medical Doctor ( )
4. Specialist (Obs/Gyn) ( )
5. Diploma in nursing ( )
6. Degree in nursing ( )
7. Master in nursing ( )

2. Age

3) Sex

1. Male ( )
2. Female ( )

4) When were you employed?

5) For how long have you worked in ANC services?

6) Have you attended training related to management pre-eclampsia/eclampsia?

1. Yes ( )
2. No ( )

**Part two: Knowledge of pre-eclampsia**

**Please tick where appropriate**

7) What is pre-eclampsia?

1. Gestation age at 20wks ( )
2. **New-onset elevated BP at 2 reading at least 4 hours apart with BP of ≥ 140 ≥ 90 mmHg and proteinuria ≥ 300 mg of protein in a 24-hour urine collection or 2+ on the dipstick in a woman without a history of proteinuria from 20 weeks of gestation age ( )**
3. Proteinuria (3+ or 300gm) ( )
4. Edema ( )

8) At what weeks of gestation age does pre-eclampsia develop?

1. Four weeks gestation age and above ( )
2. **20 weeks gestation age and above ( )**
3. Below 16weeeks gestation age ( )
4. Below 12 weeks gestation age ( )

9) What are the symptoms of Pre-eclampsia?

1. **Blurry vision and Headache ( )**
2. Diarrhea ( )
3. Pain in the abdomen ( )

10) What are the screening tests for the prediction of Pre-eclampsia?

1. **Urine Analysis ( )**
2. Blood Test ( )
3. Sputum test ( )
4. I don’t know ( )

11) What are the risk factors for developing preeclampsia?

1. **Prim gravidity and previous history of pre-eclampsia ( )**
2. Previous scar ( )
3. history of malaria during pregnancy
4. Obesity ( )

**Knowledge of the management of pre-eclampsia**

12) What are the stated drugs for controlling blood pressure? (Choose all that apply).

1. **Methyldopa ( )**
2. **Magnesium sulphate ( )**
3. Folic acid ( )
4. Diazepam ( )

14) Magnesium sulphate can be used in which category of pre-eclampsia

1. Mild pre-eclampsia ( )
2. **Severe pre-eclampsia ( )**

15) What is the loading dose of magnesium sulfate?

1. 14gm ( )
2. 10gm ( )
3. **5gm ( )**
4. 13gm ( )

16) What are the signs of magnesium sulphate toxicity (Choose all that apply).

1. **Respiratory depression and Loss of tendon reflexes ( )**
2. **Convulsion ( )**
3. Bleeding ( )

17) What is the antidote for magnesium sulphate overdose?

1. Diazepam ( )
2. **Calcium gluconate ( )**
3. Potassium ( )
4. Folic acid ( )

**Appendix 2: Skills Assessment (Jhapigo, 2017)**

| **SN** | **ACTIVITY** | **TASK (HCP able to explain)** |
| --- | --- | --- |
| 1 | Describe how the screening on danger signs of severe pre-eclampsia is conducted | - BP in the “severe” category (sBP ≥ 160 mmHg OR dBP ≥ 110 mmHg).   YES ( ) NO ( )   - Severe headache not relieved by analgesics. YES ( ) NO ( ) - Visual changes such as blurred vision   YES ( ) NO ( )   - Right upper quadrant pain.   YES ( ) NO ( ) |
| 2 | Describe how to assess for convulsion | - Rolling and bulging eyes   YES ( ) NO ( )   - Twitching of face and hand muscles YES ( ) NO ( ) - Clenching of the fists and teeth   YES ( ) NO ( )   - Violent contractions of the muscles YES ( ) NO ( ) - Foaming at the mouth YES ( ) NO ( ) - Noisy breathing   YES ( ) NO ( ) |
| 3 | Describe how to provide loading dose of magnesium sulphate | - Give MgSO4 20% solution, 4 g IV over 5–20 minutes:   YES ( ) NO ( )   - Take one 20 mL sterile syringe - Draw 8 mL (4 g) of MgSO4 50% into syringe.   YES ( ) NO ( )   - Add 12 mL of sterile water for injection to make a 20% solution.   YES ( ) NO ( )   - Follow immediately with 10 g of MgSO4 50% solution, 5 g in the upper, outer quadrant of each buttock:   YES ( ) NO ( )   - Take two 20 mL sterile syringes. Draw 10 mL (5 g) of MgSO4 50% into each syringe.   YES ( ) NO ( )   - Add 1 mL of 2% lignocaine in each syringe.   YES ( ) NO ( )   - Give deep IM injection in each buttock YES ( ) NO ( ) |
| 3 | Describe how to provide maintenance dose of magnesium sulphate | - Take one 20 mL sterile syringe, Draw 10 mL (5 g) of MgSO4 50% into syringe.   YES ( ) NO ( )   - Add 1 mL of 2% lignocaine to the syringe.   YES ( ) NO ( )   - Verify in which buttock the last MgSO4 injection was given   YES ( ) NO ( )   - Give deep IM injection in alternate   Buttock YES ( ) NO ( ) |
| 4 | Describe how to assess the magnesium sulfate toxicity | - Count respirations for 1 minute   YES ( ) NO ( )   - Check patellar reflexes.   YES ( ) NO ( )   - Insert an indwelling urinary catheter and measure urinary output.   YES ( ) NO ( ) |
| 6 | Describe how to check urine for proteinuria | - Collect a clean-catch urine sample.   YES ( ) NO ( )   - Dip the end of the dipstick into the urine. Shake off excess urine by tapping the dipstick on the side of the cup.   YES ( ) NO ( )   - Compare dipstick pad with the color chart on the dipstick container after time noted on dipstick instructions has passed   YES ( ) NO ( ) |
| 7 | Describe how to provide care during fit | - Shout for help to mobilize the team   YES ( ) NO ( )   - Turn woman onto her side to prevent aspiration   YES ( ) NO ( )   - Check for Airway, Breathing Circulation YES ( ) NO ( ) - Protect from injury YES ( ) NO ( ) - Do not put anything in her mouth YES ( ) NO ( ) |
